# Characterization data for T cell-specific Blimp-1 transgenic C57BL/6 mice

**DOI:** 10.1016/j.dib.2018.04.132

**Published:** 2018-05-08

**Authors:** Aline Yen Ling Wang, Charles Yuen Yung Loh, Shyi-Jou Chen, Huang-Kai Kao, Cheng-Hung Lin, Sheng-Hao Chuang, Chin-Ming Lee, Huey-Kang Sytwu, Fu-Chan Wei

**Affiliations:** a Center for Vascularized Composite Allotransplantation, Chang Gung Memorial Hospital, Taoyuan, Taiwan; b Division of Surgery and Interventional Science, University College London, London, United Kingdom; c Department of Pediatrics, Tri-Service General Hospital, National Defense Medical Center, Taipei, Taiwan; d Department of Plastic Surgery, Chang Gung Memorial Hospital, Taoyuan, Taiwan; e College of Medicine, Chang Gung University, Taoyuan, Taiwan; f Department of General Surgery, Chang Gung Memorial Hospital, Taoyuan, Taiwan; g Department of Microbiology and Immunology, Graduate Institute of Life Sciences, National Defense Medical Center, Taipei, Taiwan; h St Andrew's Center for Burns and Plastic Surgery, Chelmsford, United Kingdom; i Department of Microbiology and Immunology, National Defense Medical Center, Taipei, Taiwan

**Keywords:** Blimp-1, Transgenic mice, T cell

## Abstract

This article is the first to provide characterization data regarding naive C57BL/6 transgenic mice with overexpression of B lymphocyte-induced maturation protein 1 (Blimp-1) under a T cell-specific pLCK promoter. The data presented are related to phenotype, Blimp-1 overexpression levels, T cell development and T cell proliferation for Blimp-1 transgenic mice. For further Blimp-1 overexpressed T cell findings regarding skin allotransplantation, please refer to the research article “Blimp-1 prolongs allograft survival without regimen via influencing T cell development in favor of regulatory T cells while suppressing Th1” (Wang et al., 2018) [1].

**Specifications Table**

**Table:** 

Subject area	*Molecular biology and immunology*
More specific subject area	*Transplant immunology*
Type of data	*Figure and table*
How data was acquired	*Flow cytometry (BD FACSCantoII) and real-time PCR (Applied Biosystems StepOnePlus)*
Data format	*Raw and analyzed data*
Experimental factors	*The comparison of phenotype, Blimp-1 expression, lymphocyte populations and T cell proliferation between Tg(−) and Tg(+) mice*
Experimental features	*The phenotype and characterization for naive Blimp-1 transgenic C57BL/6 mice*
Data source location	*Taoyuan, Taiwan*
Data accessibility	*Data are available in this article*

**Value of the data**

• The first characterization data for T cell-specific Blimp-1 transgenic C57BL/6 mice.
• Lymphocyte proliferation data can be used for a further understanding on the Blimp-1 overexpressed T cell-mediated immunology.
• Lymphocyte population data is valuable for researchers interested in Blimp-1-modulated T cell development.

## 1 Data

**Fig. 1:**
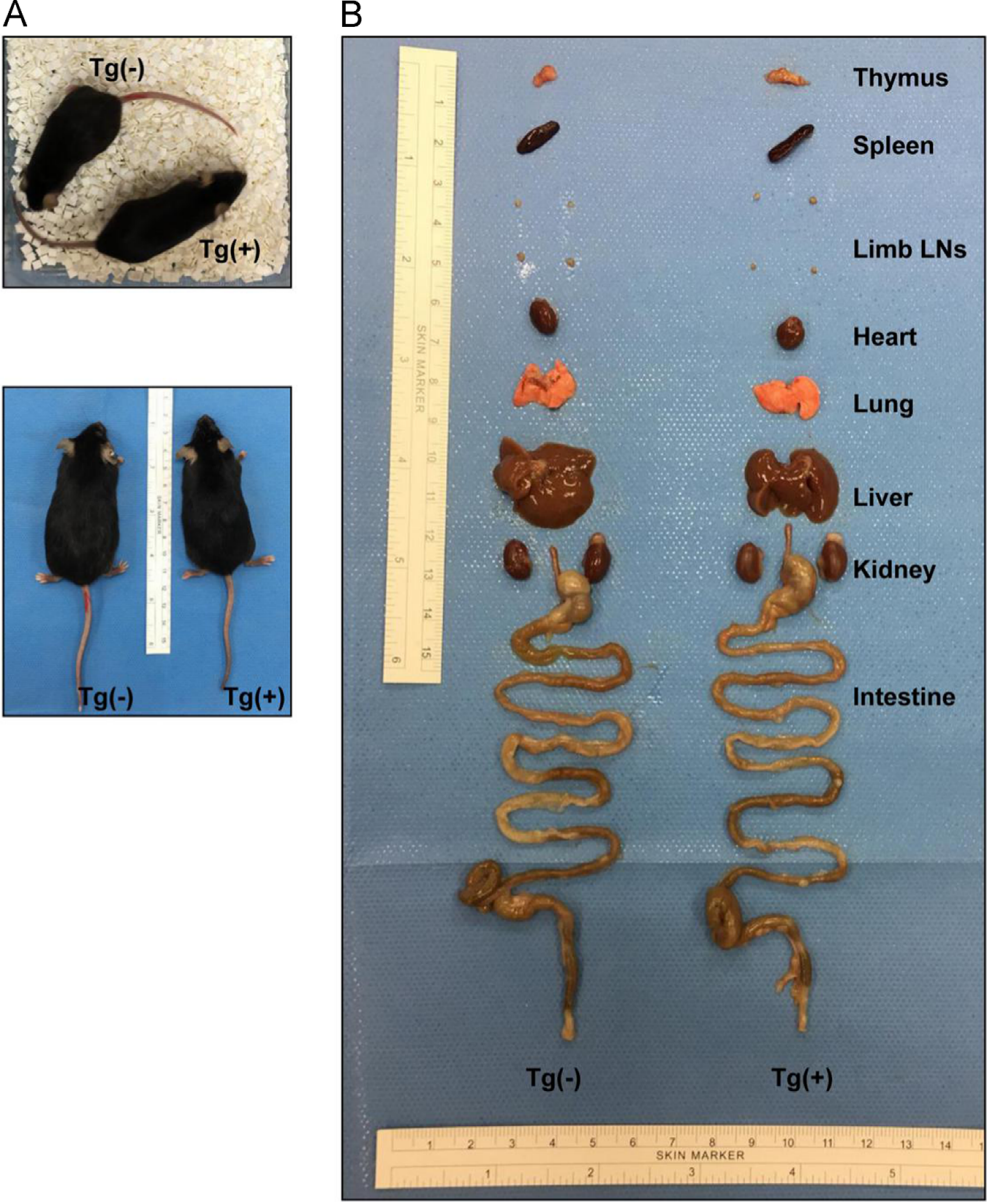
Phenotype of Blimp-1 overexpression transgenic C57BL/6 mice. (A) A side-by-side macroscopic comparison of one-year-old Tg(−) and Tg(+) mice both awake (above) and anesthetized (below). Note the similarity in size (head to tail), features, fur coat, activity and behavior. (B) Organ mapping of one-year-old Tg(−) and Tg(+) mice. Both mice had similar features in both lymphoid (thymus, spleen, axillary and groin lymph nodes) and solid organs (heart, lung, liver, kidney and intestines) as annotated. The data was collected from 3 mice in each group and from three independent experiments.

**Fig. 2:**
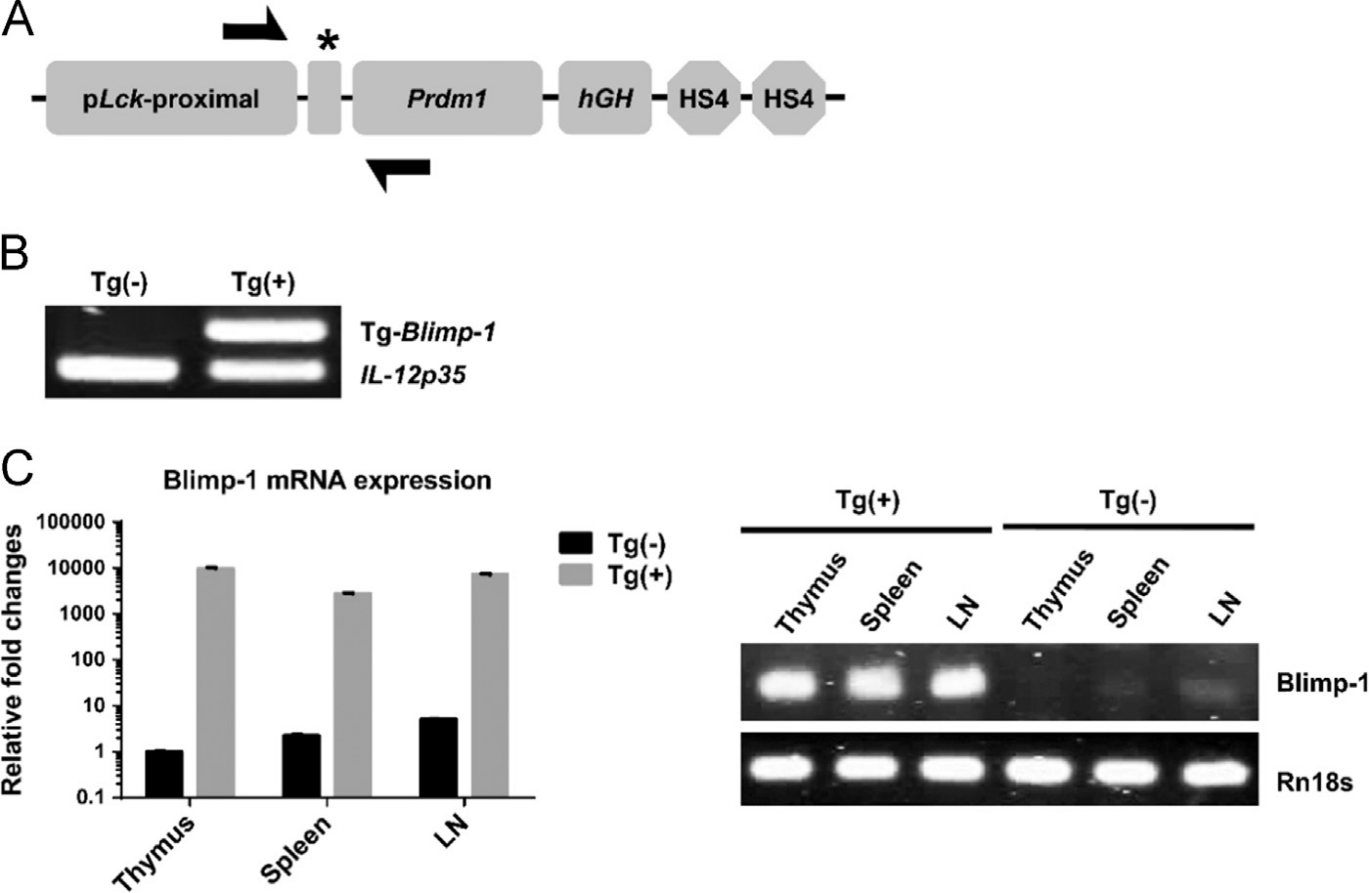
Genotyping and mRNA characterization of Blimp-1 overexpression transgenic C57BL/6 mice. (A) Schematic diagram depicting the transgene construct with a pLck-proximal driven promoter. The forward and backward design of primers identifying the transgene are also indicated. (B) PCR genotyping of Tg(+) and Tg(−) mice. Genomic DNA from Tg(+) and Tg(−) mice were obtained from tails of mice and identified using PCR with the specific primers designed in A. Tg(+) mice indicated positive for the Tg-Blimp-1 band on gel electrophoresis. (C) Characterization of Blimp-1 mRNA expression in lymphoid organs. Levels of Blimp-1 mRNA expression were quantified using real-time PCR in the thymus, spleen and lymph nodes of naïve twelve-week-old Tg(+) and Tg(−) mice. Thymus, spleen and lymph nodes from Tg(+) mice were analyzed with reverse transcriptase-PCR and showed positive bands for Blimp-1 on gel electrophoresis with Rn18s bands as an internal control. The data was collected from 6 mice in each group and from six independent experiments.

**Fig. 3:**
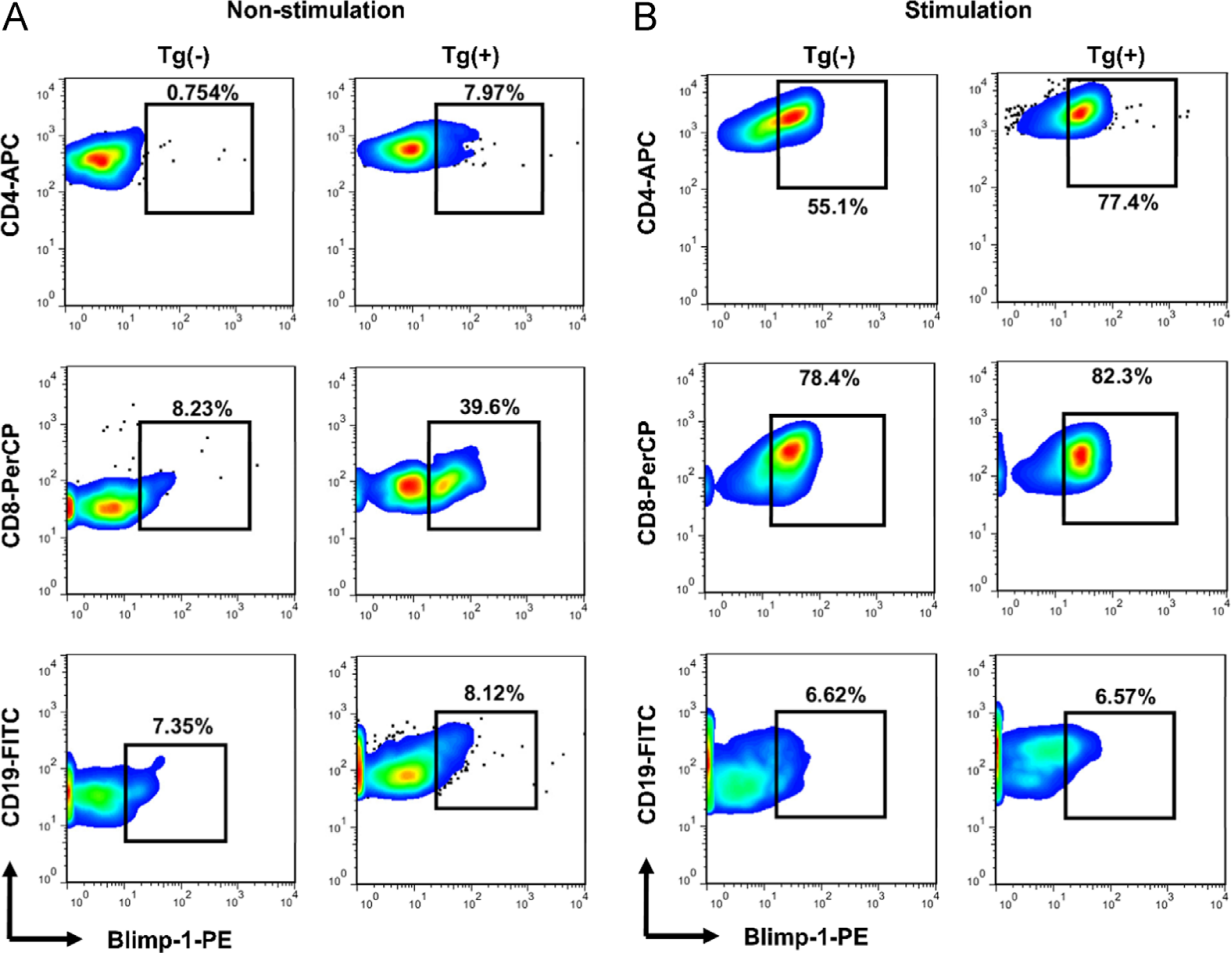
Quantification of Blimp-1 protein expression in naïve Tg(+) and Tg(−) mice. (A) Blimp-1 protein levels of CD4^+^, CD8^+^ and CD19^+^ cells under unstimulated conditions. Lymphocytes from the spleen and lymph nodes of twelve-week-old Tg(+) and Tg(−) mice were harvested and stained for CD4^+^, CD8^+^, CD19^+^ and Blimp-1. (B) Blimp-1 protein levels of CD4^+^, CD8^+^ and CD19^+^ cells under stimulation. Lymphocytes from the spleen and lymph nodes of Tg(+) and Tg(−) mice were harvested and stimulated with anti-CD3 (1 µg/ml) for 3 days. The cells were then stained for CD4^+^, CD8^+^, CD19^+^ and Blimp-1 and analyzed using flow cytometry. The levels of Blimp-1 in each cell type were gated and analyzed using flow cytometry.

**Fig. 4:**
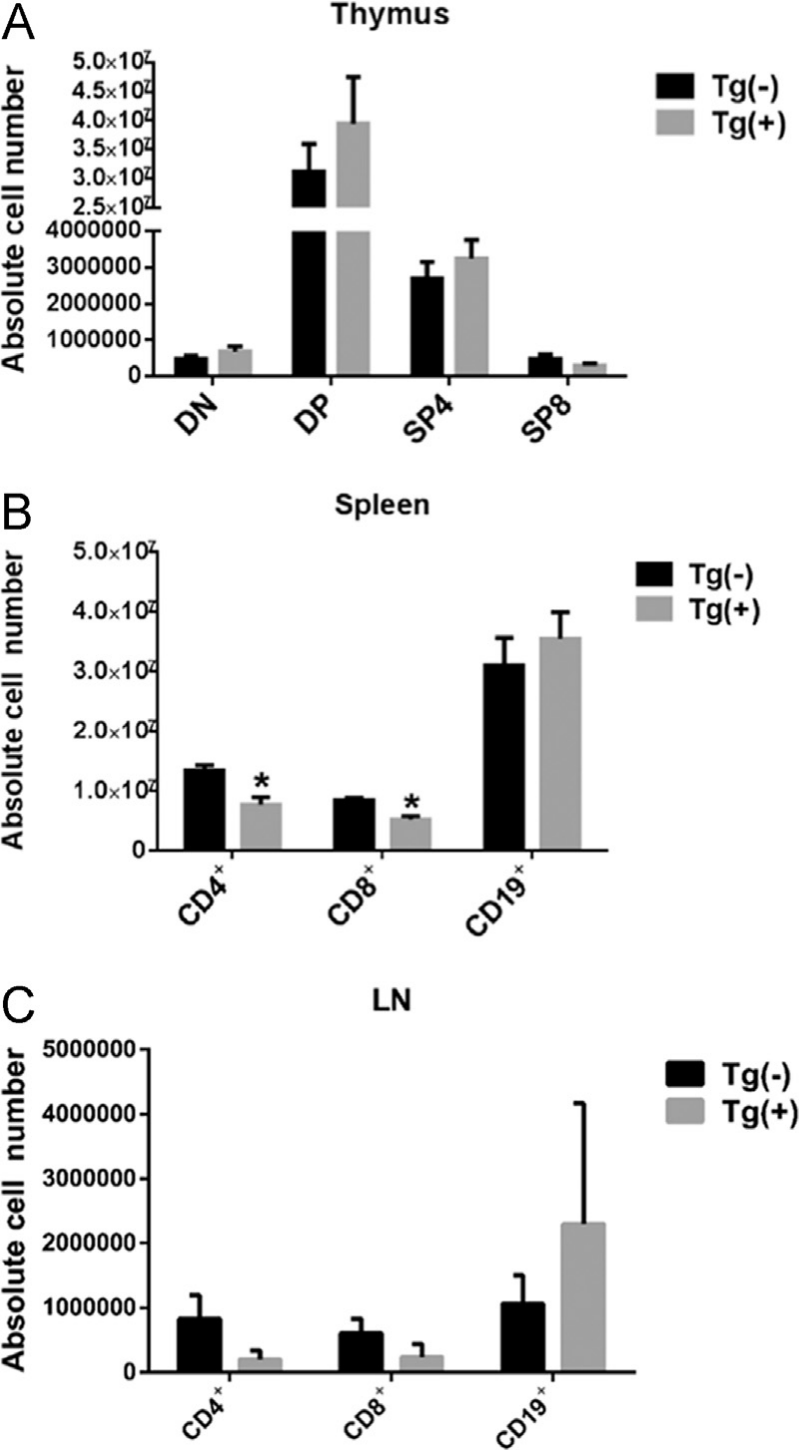
T cell development in naïve Tg(+) and Tg(−) mice. (A) The thymus from six to eight-week-old Tg(+) and Tg(−) mice were harvested and stained for CD4^+^ and CD8^+^. (B, C) The spleen and lymph nodes from Tg(+) and Tg(−) mice were harvested and stained for CD4^+^, CD8^+^ and CD19^+^. The absolute cell number of each cell type was quantified using flow cytometry. The data was collected from 5 mice in each group and from five independent experiments. The statistical data was represented as a mean ± SEM. *P < 0.05.

**Fig. 5:**
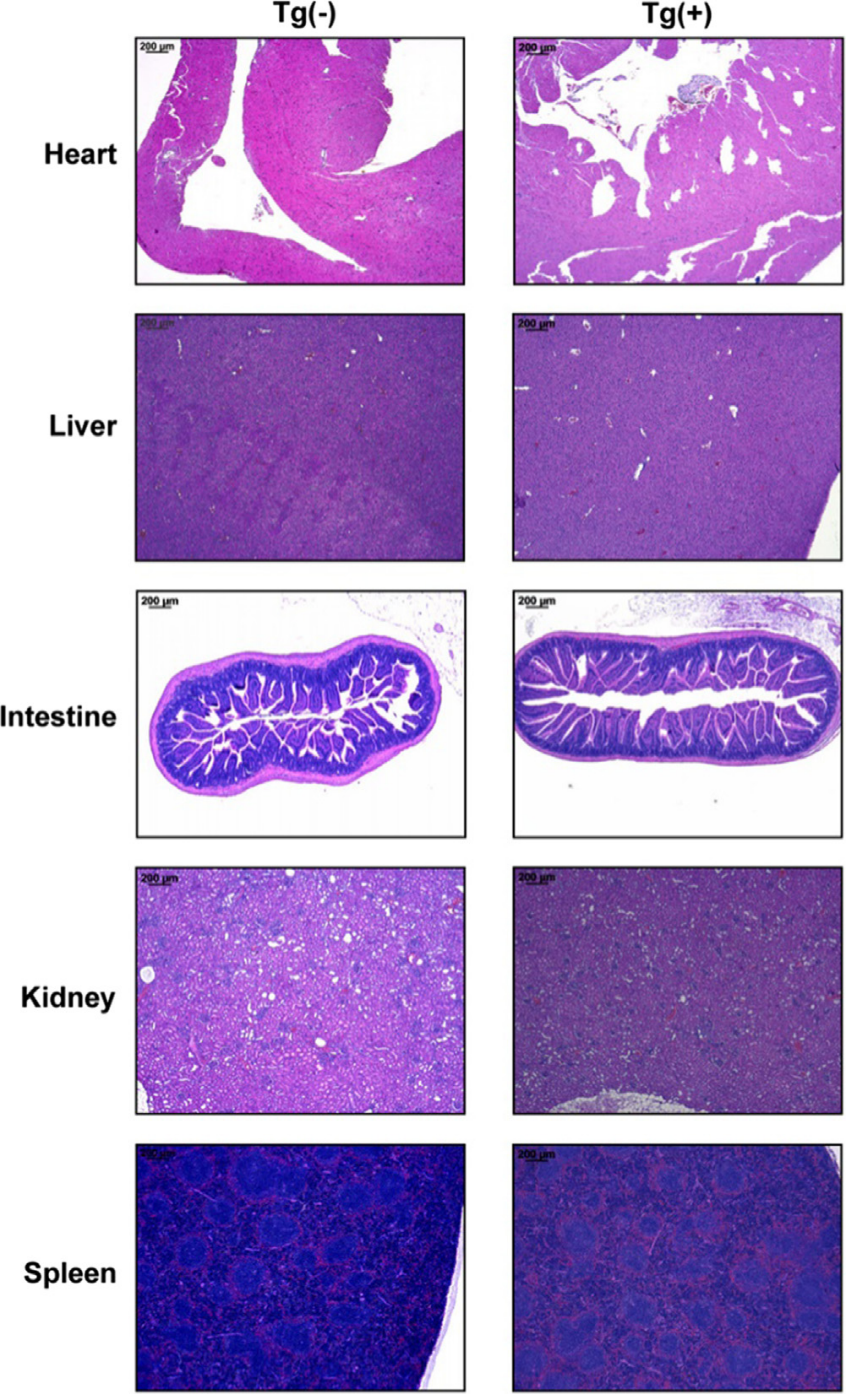
Pathological evaluation of naïve one-year-old Tg(+) and Tg(−) mice. H&E pathological evaluation of various organs from mice in both groups. Note the similar normal tissue architecture in naïve Tg(+) and Tg(−) mice. The data was collected from 3 mice in each group and from three independent experiments.

**Fig. 6:**
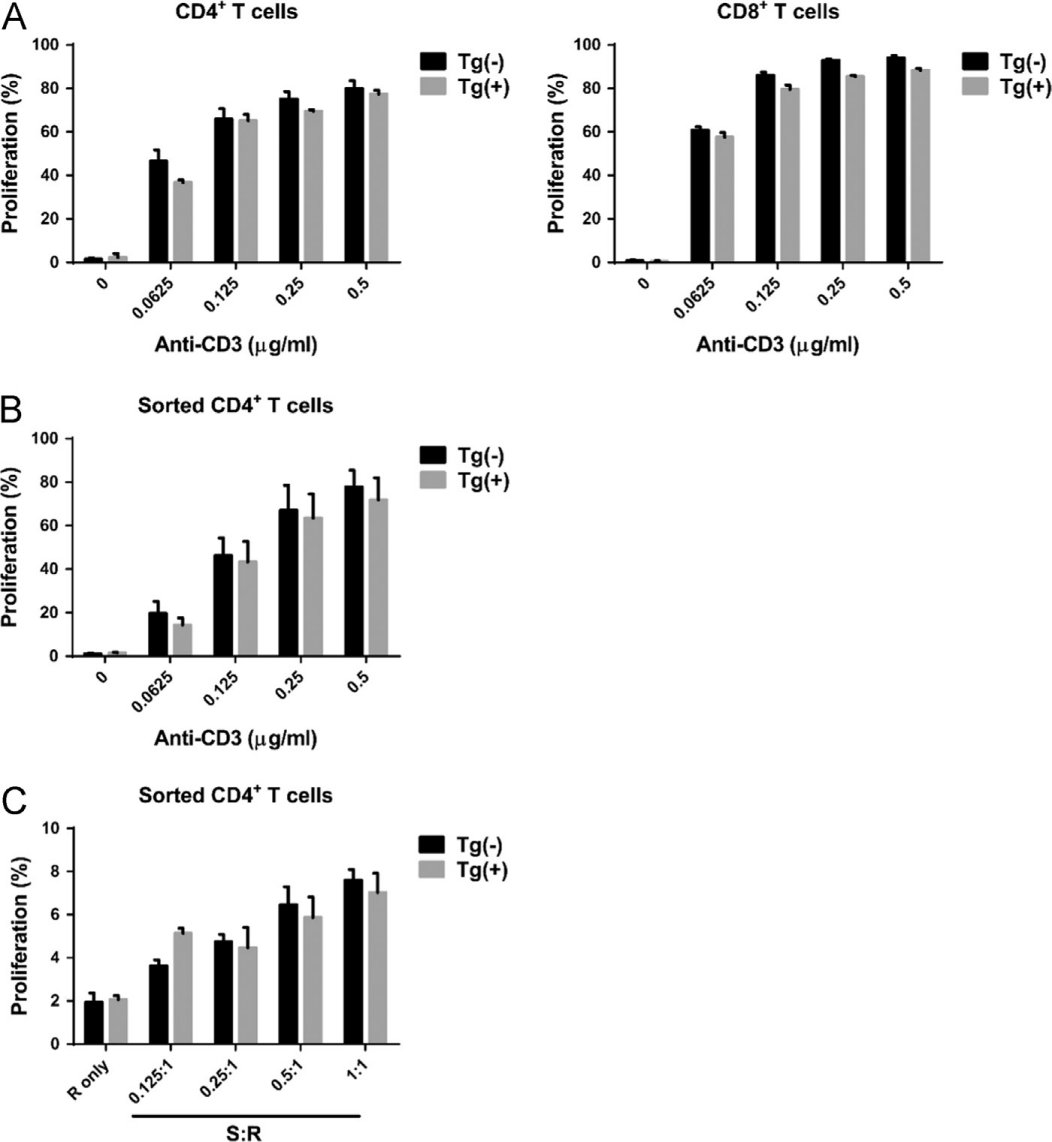
Lymphocytic proliferation in naïve Tg(+) and Tg(−) mice. (A) CD3 receptor-dependent lymphocytic proliferation. Naive lymphocytes were harvested from splenocytes and LNs of eight to ten-week-old Tg(+) and Tg(−) mice. Lymphocytes were stimulated with varying concentrations of anti-CD3 mAb for 3 days. The CD4^+^ and CD8^+^ T cell proliferation were evaluated by VPD-450 dye dilution using flow cytometry. (B) CD3 receptor-dependent sorted CD4^+^ T cell proliferation. Naive sorted CD4^+^ T cells were harvested and purified from the LNs of eight to ten-week-old Tg(+) and Tg(−) mice using autoMACS with a purity of more than 90%. Methods used are similar to (A). (C) The proliferation of sorted CD4^+^ T cells in response to alloantigens. Mix lymphocyte reaction assay was used to evaluate the CD4^+^ T cell alloreactivity. S represents stimulators whereas R the responders. Sorted CD4^+^ T cells (responder) from splenocytes and LNs of eight to ten-week-old Tg(+) and Tg(−) were labeled with VPD-450. They were then cocultured with irradiated CD90.2-depleted lymphocytes from BALB/c donor mice (stimulator) for 3 days. Analysis and quantification was performed using flow cytometry. Similar results were obtained from three independent experiments and represented as a mean ± SEM.

**Fig. 7:**
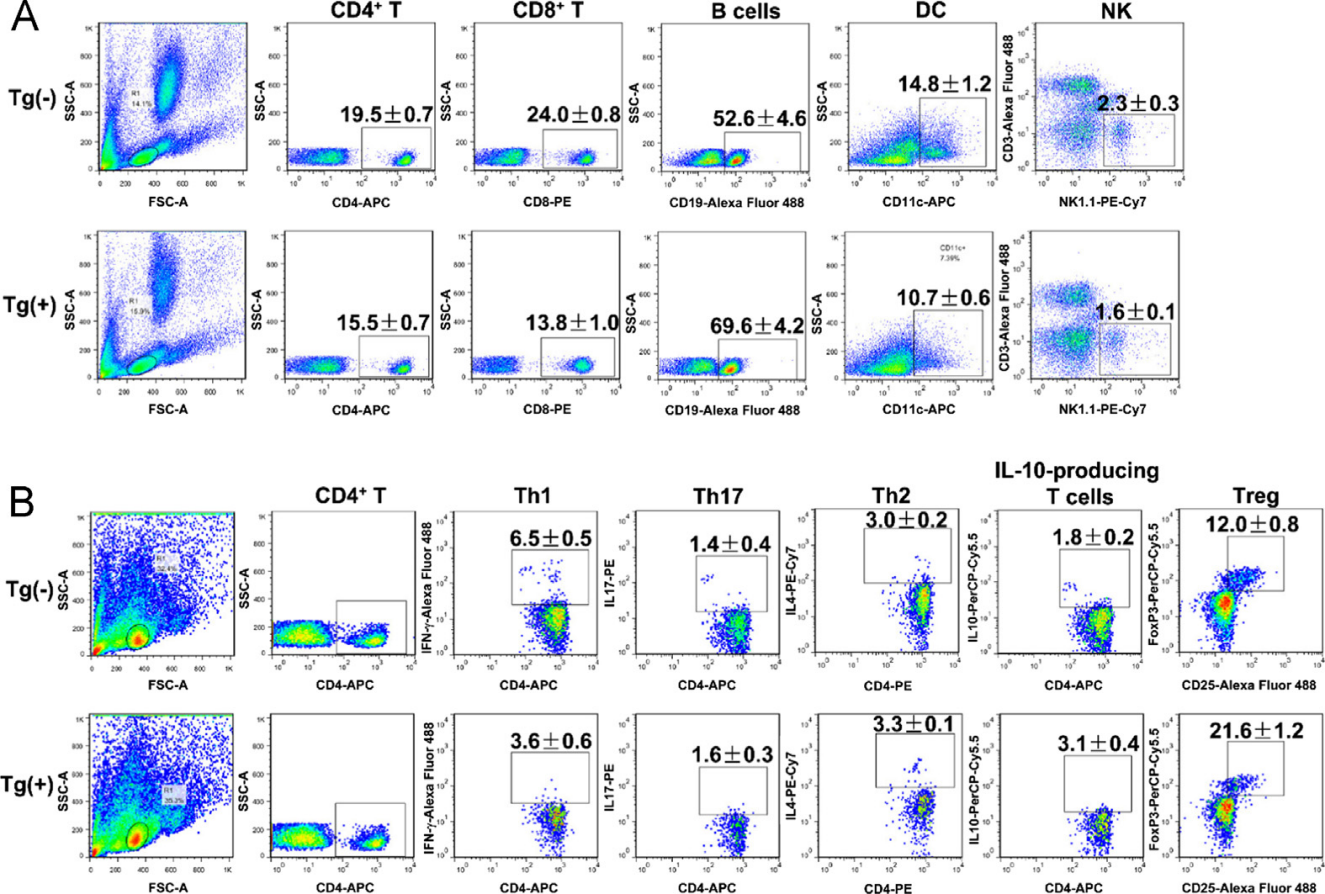
Pseudocolor plots of flow cytometry for blood lymphocyte and CD4^+^ T cell subsets in both skin transplanted mice. (A) Blood lymphocyte subsets. Blood was taken from each group at POD 10 for lymphocyte subset analysis. The data was collected from 10 mice in each group. (B) Blood CD4^+^ T cell subsets. Blood was taken from each group at POD 10 for CD4^+^ T cell subset analysis. Intracellular staining technique, using the following stains, was performed: Th1 cells using CD4^+^IFN-γ^+^, Th17 cells using CD4^+^IL-17^+^, Th2 using CD4^+^IL-4^+^, IL-10-producing T cells using CD4^+^IL-10^+^ and lastly Treg using CD4^+^CD25^+^FoxP3^+^ markers. The data was collected from 6 mice in each group.

**Fig. 8:**
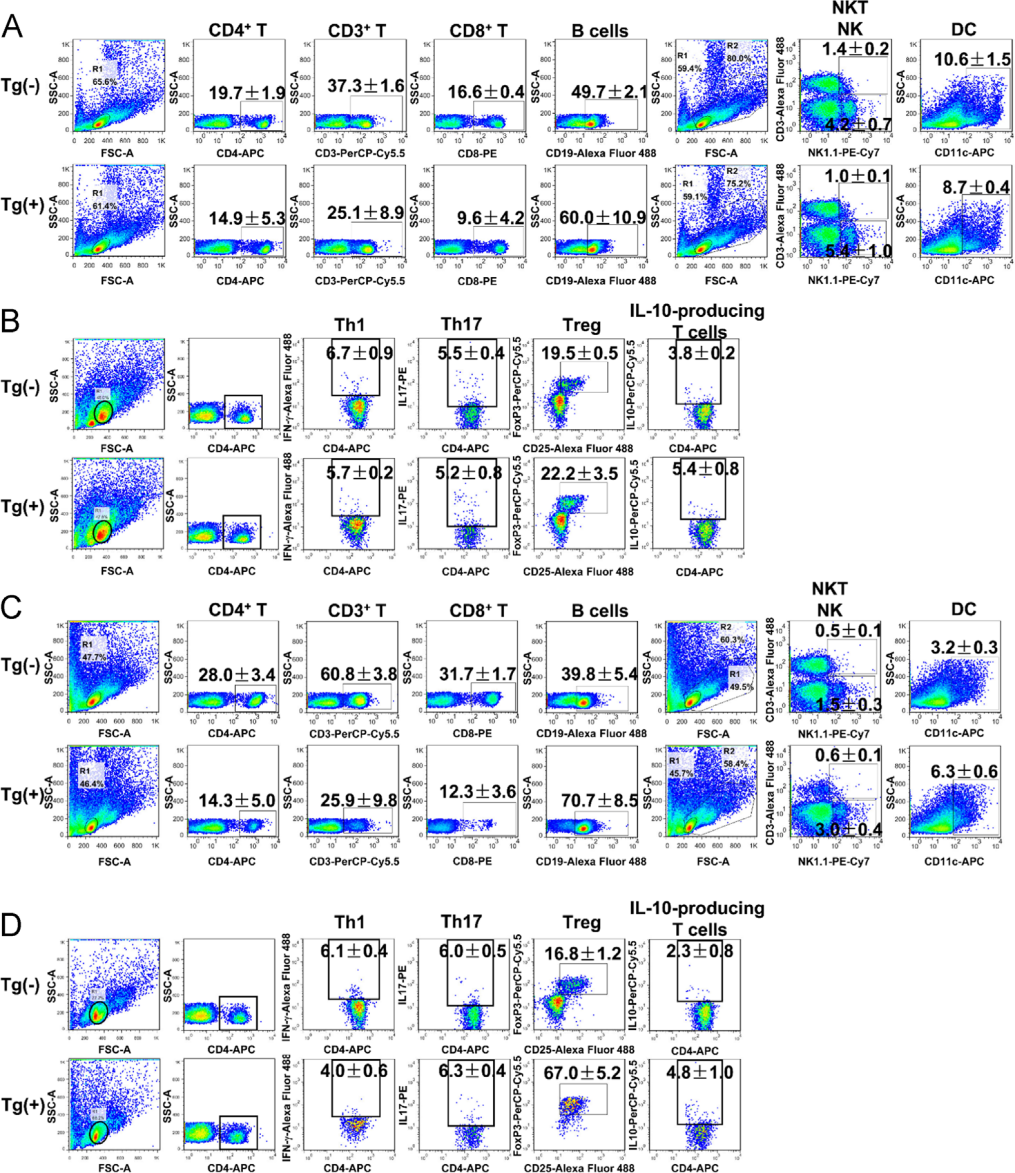
Pseudocolor plots of flow cytometry for lymphocyte and CD4^+^ T cell subsets from spleen and LNs in both skin transplanted mice. (A, C) Lymphocyte percentages in spleen and LNs respectively. Spleens and LNs were harvested from the skin allografted mice in each group (*N* = 5) at POD 10 for lymphocyte subset analysis. (B, D) Percentage of inflammatory and anti-inflammatory cell subsets in spleen and lymph nodes of Tg(+) and Tg(−) mice respectively. Spleen and lymph nodes were harvested from each group at POD 10 for CD4^+^ T cell subset analysis. The cells were stained for CD4^+^IFN-γ^+^ Th1 cells, CD4^+^IL-17^+^ Th17 cells, CD4^+^CD25^+^FoxP3^+^ Tregs and IL-10-producing T cells using CD4^+^IL-10^+^. The data was collected from 3–6 mice in each group.

This article provides detailed characterization data of Blimp-1 transgenic mice for macroscopic phenotype and organ comparison of one-year-old Tg(−) and Tg(+) mice (Fig. 1A and B). The schematic diagram depicting the transgene construct with a pLck-proximal driven promoter, PCR genotyping and mRNA overexpression of Blimp-1 are indicated in Fig. 2A, B and C, respectively. Table 1 shows the primer sequence information used in the PCR experiments of Fig. 2. The Blimp-1 protein overexpression levels of T cells under unstimulated and stimulated conditions are shown in Fig. 3A and B. T cell development in thymus, spleen and lymph node was examined between naïve Tg(+) and Tg(−) mice (Fig. 4A, B and C). Pathological evaluation of various organs from naïve one-year-old mice in both groups is presented in Fig. 5 and Table 2. CD3-dependent lymphocytic and sorted CD4^+^ T cell proliferation in both naïve mice are presented in Fig. 6A and B. Fig. 6C evaluates CD4^+^ T cell alloreactivity using mix lymphocyte reaction in both naive mice. Fig. 7A and B show blood lymphocyte and CD4^+^ T cell subsets in both skin transplanted mice. Fig. 8 shows lymphocyte and CD4^+^ T cell subsets from spleen (Fig. 8A and B) and LNs (Fig. 8C and D) in both skin transplanted mice. For further Blimp-1 overexpressed T cell findings regarding skin allotransplantation, please refer to the research article “Blimp-1 prolongs allograft survival without regimen via influencing T cell development in favor of regulatory T cells while suppressing Th1” [1].

**Table 1:** Primer sequence information used in the PCR experiments.

**Primer**	**Sequence information**	**Objective**
**Prdm1-F**	5′-TAATGAAGAGGGACAGGTACCCTC-3′	PCR genotyping
**Prdm1-R**	5′-TCCAAAGCCGTGTAAAGTAGACTG-3′	PCR genotyping
**Blimp-1**	Mm00476128_m1	TaqMan gene expression assays

F, forward primer; R, reverse primer.

**Table 2:** One year H&E pathological evaluation in organs of naïve Tg(−) and Tg(+) mice.

	**Tg(−) *N* = 3**	**Tg(+) *N* = 3**
**Heart**	–	–
**Liver**	–	–
**Intestine**	–	–
**Kidney**	–	–
**Spleen**	–	–

“–” represents negative pathological findings on microscopic examination of various organs.

## 2 Experimental design, materials and methods

### 2.1 Mice

T cell-specific Blimp-1 transgenic C57BL/6 mice were kindly provided by Professors HK Sytwu and SJ Chen of the National Defense Medical Center, Taiwan. Transgenic mice were overexpressed with Blimp-1 under a T cell-specific pLCK promoter. All murine procedures were carried out in full compliance with the recommendations in the Guide for the Care and Use of Laboratory Animals of the Chang Gung Memorial Hospital Animal research guidelines. Animal protocols were approved by the Committee on the Ethics of Animal Experiments of the Chang Gung Memorial Hospital (CGMH) in Taiwan and Institutional Animal Care and Use Committees (IACUC) of CGMH in Taiwan under permit numbers IACUC2014032502 and IACUC2016031109.

### 2.2 Flow cytometry analysis

Lymphocytes were harvested from Tg(+) and Tg(−) mice and stained with antibodies for cell surface markers. Antibodies such as CD4, CD8, CD3, CD19, CD11c, NK1.1, CD25, IFN-γ, IL-17, IL-4, IL-10, FoxP3, Blimp-1 for flow cytometry were purchased from eBioscience (San Diego, CA) and BD Biosciences. For intracellular cytokine staining, cells were stimulated with phorbol 12-myristate 13-acetate (20 ng/ml), ionomycin (1 μg/ml), and monesine (4 μM) for 4 h and intracellular cytokine staining was performed [2].

### 2.3 Real-time polymerase chain reaction (qPCR)

The expression of mRNA for Blimp-1 in the lymphoid organs was analyzed with TaqMan gene expression assays (Thermo Fisher Scientific, Waltham, MA). The expression was normalized to that of Rn18s [3].

### 2.4 Lymphocyte proliferation

Lymphocytes were harvested from naive transgenic mice and stained with Violet Proliferation Dye 450 (VPD450) (BD Biosciences, San Jose, CA). VPD450 labeled lymphocytes (2 × 10^5^ cells per well) were stimulated with different concentrations of bound anti-CD3 antibodies for three days. T cell proliferation was then assessed by flow cytometry.

### 2.5 Mixed lymphocyte reaction

Sorted CD4^+^ T cells from naive Tg(+) and Tg(−) mice were stained with VPD450 proliferation dye and responders (2 × 10^5^ cells) were co-cultured with stimulator irradiated donor lymphocytes which were irradiated with 2500 rads in a 96-well round bottom plate for three days. T cell proliferation was assessed by flow cytometry.

## References

[1] Wang A.Y.L., Loh C.Y.Y., Chen S.J., Kao H.K., Lin C.H., Chuang S.H., Lee C.M., Sytwu H.K., Wei F.C. (2018). Blimp-1 prolongs allograft survival without regimen via influencing T cell development in favor of regulatory T cells while suppressing Th1. Mol. Immunol..

[2] Lao W.W., Wang A.Y.L., Ramirez A.E., Cheng H.Y., Wei F.C. (2014). A new rat model for orthotopic abdominal wall allotransplantation. PRS GO.

[3] Loh C.Y.Y., Wang A.Y.L., Kao H.K., Cardona E., Chuang S.H., Wei F.C. (2016). Episomal induced pluripotent stem cells promote dunctional recovery of transected murine peripheral nerve. PLoS One.

